# Artificial blood-feeding of phlebotomines (Diptera: Psychodidae: Phlebotominae): is it time to repurpose biological membranes in light of ethical concerns?

**DOI:** 10.1186/s13071-022-05511-4

**Published:** 2022-10-31

**Authors:** Yetsenia del Valle Sánchez Uzcátegui, Eduardo José Melo dos Santos, Edilson Rodrigues Matos, Fernando Tobias Silveira, Thiago Vasconcelos dos Santos, Marinete Marins Póvoa

**Affiliations:** 1 Programa de Pós Graduação em Biologia de Agentes Infecciosos e Parasitários, Instituto de Ciências Biológicas, Universidade Federal do Pará, Belém, Brazil; 2 Seção de Parasitologia, Instituto Evandro Chagas, Ananindeua, Brazil; 3 Departamento de Biología, Facultad de Ciencias, Universidad de Los Andes, Mérida, Venezuela; 4 Genetics of Complex Diseases Laboratory, Universidade Federal do Pará, Belém, Brazil; 5 Laboratório de Pesquisa Carlos Azevedo, Universidade Federal Rural da Amazônia, Belém, Brazil

**Keywords:** Phlebotominae, *Leishmania*, Membrane, Artificial feeding, Chick, Skin, Experimental infection

## Abstract

**Background:**

The aims of the present study were to evaluate and compare the efficacy of blood-feeding in phlebotomines through industrially processed membranes from the small intestine of pigs (used for the production of commercial sausages) and the skin of euthanized chicks.

**Methods:**

Laboratory-bred *Lutzomyia longipalpis* and different field-caught phlebotomine species were subjected to the artificial feeding systems under similar conditions. Paired tests were performed using the control (skin from euthanized chicks) and test membranes (pig small intestine). The feeding rates were compared by paired* t*-test, and Pearson correlation was used to examine the relationship between the thickness of the membranes and feeding rate.

**Results:**

The feeding rate was greater with the test membrane than with the control membrane for *L. longipalpis* (*t*-test, *t* = −3.3860, *P* = 0.0054) but not for the most frequent field-caught species, *Nyssomyia antunesi* (*t*-test, *t* = 0.7746, *P* = 0.4535). The average thicknesses of the control and test membranes were 184 ± 83 µm and 34 ± 12 µm, respectively (Mann–Whitney *U*-test, *U* = 0.00, *Z* = 2.8823, *P* = 0.0039); however, there was no correlation between feeding rate and membrane thickness. A moderate positive correlation was observed between the number of phlebotomines that fed and the total number of phlebotomines in the cage for each type of membrane and for each species.

**Conclusions:**

The test membrane is a viable alternative for the artificial blood-feeding of phlebotomines, and is thus a potential substitute for the skin of animals that are euthanized for this purpose. Feeding rate was independent of membrane thickness.

**Graphical Abstract:**

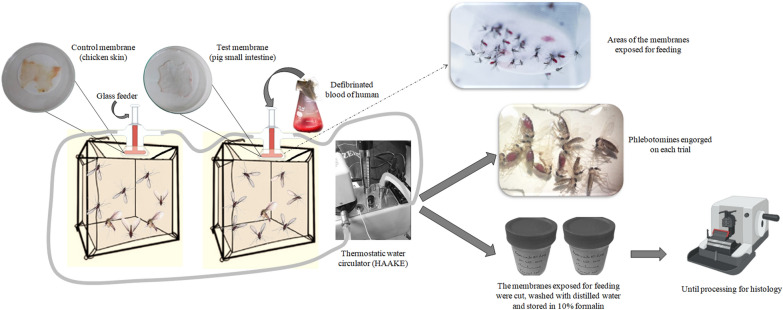

## Background

Phlebotomines (Diptera: Psychodidae: Phlebotominae) are medically important haematophagous insects due to their recognized role in the transmission of several pathogens, and in particular *Leishmania* protozoa, the causal agents of leishmaniosis [1]. Adult phlebotomines of both sexes feed on carbohydrates, which they use as an energy source; only the females feed on blood, which provides essential proteins for the development and maturation of eggs [2–5]. Thus, the transmission of *Leishmania* parasites is carried out by females, and requires at least two blood meals, of which the first is from an infected reservoir host [2, 6–8].

A supply of blood is required for colony maintenance [3, 4, 9] and parasite-vector interaction studies of phlebotomines [4, 9–11]. The insects are commonly fed by in vivo methods using animals [3, 12] or in vitro methods using artificial feeding systems [4, 11–14] with natural or synthetic membranes [11, 15]. Achieving adequate membrane feeding of phlebotomines is difficult. It is necessary to reproduce natural conditions for each species of phlebotomine, and success is dependent on the effect of several factors, such as blood/membrane origin, blood/environment temperature, humidity and the presence of chemical/textural stimuli [4, 15]. According to Fatemi et al. [11], it is more difficult to rear phlebotomines than other haematophagous insects.

Several membranes have been tested for the artificial blood-feeding of phlebotomines [3, 11–13, 15–20]. Since the 1970s, chick skin has been recognized as the gold standard for this purpose [16], and is widely used [3, 4, 9, 11, 15, 16, 21–23]. However, due to controversy concerning reproducibility [3] and ethical concerns regarding animal welfare, methods requiring vertebrate euthanasia have been questioned and progressively discouraged [17, 24]. Pig intestine membranes obtained from industrially processed sausages have already been successfully used with *Psathyromyia shannoni* [18]. However, these have never been tested with laboratory-bred *Lutzomyia longipalpis*, one of the most medically important and widely studied phlebotomine species, which represents 88% of the phlebotomine colonies registered in the Americas [25], or with field-caught species. Thus, the aim of the present study is to evaluate the artificial blood-feeding of laboratory-bred *L. longipalpis* and different field-caught phlebotomine species through an industrially processed membrane from the small intestine of pigs which is used in the production of commercial sausages.

## Methods

### Phlebotomines

The laboratory-bred phlebotomines were specimens of *L. longipalpis* obtained from a closed colony (strain Cametá F38) maintained in the Ralph Lainson Leishmaniasis Laboratory, Instituto Evandro Chagas, Belém, Brazil. For the trials, 2- to 3-day-old adult female specimens were used [3, 26], which were confined to a 16-cm^3^ nylon cage [858 squares/cm^2^, squares of 265 (± 19) µm by 291 (± 14) µm and 158 (± 10) µm by 256 (± 24) µm]. The cage was placed inside a plastic bag containing moist cotton to maintain the relative humidity at 80 ± 10% [15, 21]. The insects were supplied with 10% glucose solution ad libitum [3] until 24 h before the trials [12, 27].

Phlebotomines were captured from the field with Centers for Disease Control and Prevention (CDC) light traps placed at 1.5 m above ground level (*n* = 2 CDC traps) and in the canopy at 20 m above ground level (*n* = 2 CDC traps), which operated from 6:00 p.m. to 6:00 a.m. for seven nights. The phlebotomines were captured in February and March 2021 in the Bosque Rodrigues Alves–Jardim Zoobotânico da Amazônia, an urban park in Belém, Brazil, in which the phlebotomine fauna had been previously characterized [28]. The phlebotomines were visually screened from other insects, aspirated from the primary cage in the field, and transported immediately to the laboratory under the environmental conditions given above. After subjecting the field-caught phlebotomines to the procedure described above for laboratory-bred phlebotomines, they were immediately transferred to a secondary nylon cage. Captured females found to be engorged, gravid or semi-gravid were excluded from the experiments.

### Membrane feeding

The control membranes comprised skin of 2- to 5-day-old chicks that had been euthanized according to the guidelines of the National Council for Animal Experimentation Control, Brazil (normative resolution no. 37/2018—guideline for the practice of euthanasia), and processed according to McMahon [29] and Ward et al. [16]. The test membranes comprised pig small intestine that had been removed from industrially processed sausages (Frimesa Cooperativa Central, Brazil). Prior to use, the intestines were opened longitudinally, washed with distilled water and rubbed with gauze to remove the remains of the sausage stuffing. They were maintained at 4 °C in sterile water for 72 h and subsequently washed every 24 h to remove excess condiment, salts and other impurities [18]. Smooth and uniform fragments were selected for use. Pieces (4 × 4 cm) were cut from the fragments, spread flat and wrapped in aluminum foil for storage at −20 °C until use [17].

Membrane (serous layer on the outside) was fixed with insulating tape over the open end (3 cm diameter) of a glass chamber [16, 17], which provided a feeding surface area of 7.1 cm^2^. Comparative assays using paired control and test membranes were performed under the same conditions, with approximately 100–150 specimens in each nylon cage for both laboratory-bred *L. longipalpis* and different field-caught phlebotomine species; the latter were taxonomically determined after the trials in accordance with Galati [30].

The artificial feeding system consisted of two interconnected glass chambers, i.e. one with the test membrane and the other with the control membrane, maintained at 36.5 ± 1 °C in a thermostatic water circulator (Haake Technik, Germany) [24, 31]. For the comparative trials, the laboratory-bred and field-caught phlebotomines were exposed to the paired membranes and allowed to feed for 2 h [3, 22]. The membrane surfaces of the glass chambers were positioned horizontally and pressed against the top of the nylon cage [17]. Each chamber was filled with 4 mL of blood that had been collected from a unique human volunteer (YVSU), mechanically defibrinated, and filtered through gauze. During the experiment, the cages were wrapped in black cloth to stimulate feeding [27]. Every 20 min, the membranes were moistened with distilled water to re-establish humidity and plasticity, and the blood shaken to avoid cell sedimentation.

After each experiment, the circular areas of the membranes exposed for feeding were cut out, washed with distilled water and stored in 10% formalin until processing for histological examination [32].

### Histology of the biological membranes

Five- to 10-mm membrane fragments were dissected under a stereomicroscope and fixed in Davidson’s solution for 24 h before being processed using standard techniques for embedding in paraffin [33, 34]. Two 5-μm serial sections were prepared [32] using a Microm HM 315 microtome (Microm, Walldorf, Germany), deparaffinized and stained on glass slides with haematoxylin–eosin [35].

The membrane fragments on the prepared slides were visualized with an Axioscope 5 microscope (Zeiss, Germany) and photographed with an Axiocam 506 camera system (Zeiss) for comparative histology. ZEN (blue edition) version 3.4 software (Zeiss, Germany) was used to measure the thickness of the membranes. A total of six samples were used for each membrane type [36]. The average thickness of the membranes was determined by measuring four sites in each section. Each site was continuous for at least 100 μm in length and had intact bedding [37].

### Data analysis

The feeding rate, which corresponds to the percentage of engorged phlebotomines in each trial [3, 4], was calculated for the laboratory-bred *L. longipalpis* and the field-caught species at a consistent sample size. Paired, comparative trials were replicated until statistical reproducibility was ensured [3]. Feeding rates were analysed using Student’s *t*-test with BioEstat 5.3 software (Instituto Mamirauá, Brazil) [38]. The thickness of the membranes was compared between groups using the Mann–Whitney test with Sigmaplot 12.5 software (Systat Software, USA). The rate of phlebotomines that fed was correlated with the number of phlebotomines in the cage using Pearson’s correlation test. Pearson’s correlation test was also carried out using BioEstat 5.3 software [38] to compare the proportion of *L. longipalpis* that fed with the thickness of each type of membrane. In all cases, the normality of the data was confirmed using the Shapiro–Wilk test, and a significant difference at* p* ≤ 0.05 was considered to indicate a 95% confidence interval.

## Results

A total of 1201 specimens belonging to 12 species were captured and used in the trials. *Nyssomyia antunesi* was the most frequent among the captured species (72.6%) (Table 1) and the only field-caught species for which the sample size was considered sufficient for statistical analysis. Two species, *Nyssomyia antunesi* and *Bichromomyia flaviscutellata*, fed through the control membrane, with average feeding rates of 1.7 ± 2.0% and 0.6 ± 1.2%, respectively, while four species, *N. antunesi*, *B. flaviscutellata*, *Trichophoromyia ubiquitalis* and *Pressatia choti*, fed through the test membrane, with average feeding rates of 1.0 ± 1.4%, 0.3 ± 0.6%, 0.3 ± 0.8% and 0.4 ± 0.9%, respectively (Table 2).

**Table 1:** Phlebotomine species captured in February and March 2021 in the Bosque Rodrigues Alves—Jardim Zoobotânico da Amazônia, Belém, Brazil and used in the comparative artificial feeding trials

Species	Males	Females	Total	%
*Nyssomyia antunesi*	748	124	872	72.6
*Trichophoromyia brachipyga*	79	65	144	12.0
*Bichromomyia flaviscutellata*	46	31	77	6,4
*Trichophoromyia ubiquitalis*	26	38	64	5.3
*Micropygomyia trinidadensis*	4	7	11	0.9
*Viannamyia furcata*	4	4	8	0.7
*Pressatia choti*	2	6	8	0.7
*Brumptomyia avellari*	5	0	5	0.4
*Micropygomyia rorotaensis*	2	2	4	0.3
*Psathyromyia bigeniculata*	2	2	4	0.3
*Viannamyia tuberculata*	0	2	2	0.2
*Evandromyia monstruosa*	2	0	2	0.2
Total	920	281	1201	100

**Table 2:** Feeding rates of phlebotomine females captured in February and March 2021 in the Bosque Rodrigues Alves–Jardim Zoobotânico da Amazônia in the comparative assays with the control and test membranes

Trials	Control (chick skin)				Test (pig small intestine)					
	*Nyssomyia antunesi*	*Bichromomyia flaviscutellata*	Other species	*n*	*N. antunesi*	*B. flaviscutellata*	*Trichophoromyia ubiquitalis*	*Pressatia choti*	Other species	*n*
1	4/76 (5%)	0/0 (0%)	0/0 (0%)	76	1/107 (1%)	1/1 (100%)	0/0 (0%)	0/0 (0%)	0/2 (0%)	110
2	2/58 (3%)	1/2 (50%)	0/9 (0%)	69	1/74 (1%)	0/0 (0%)	0/4 (0%)	0/0 (0%)	0/6 (0%)	84
3	0/22 (0%)	1/5 (20%)	0/6 (0%)	33	0/25 (0%)	0/9 (0%)	0/1 (0%)	0/0 (0%)	0/6 (0%)	41
4	0/31 (0%)	0/2 (0%)	0/13 (0%)	46	0/32 (0%)	0/0 (0%)	1/4 (25%)	0/1 (0%)	0/10 (0%)	47
5	0/37 (0%)	0/5 (0%)	0/24 (0%)	66	1/60 (2%)	1/5 (20%)	0/2 (0%)	0/0 (0%)	0/6 (0%)	73
6	2/102 (2%)	0/2 (0%)	0/7 (0%)	111	3/68 (4%)	0/5 (0%)	0/1 (0%)	0/0 (0%)	0/5 (0%)	79
7	1/32 (3%)	0/6 (0%)	0/8 (0%)	46	0/25 (0%)	0/3 (0%)	0/3 (0%)	1/1 (100%)	0/9 (0%)	41

**Fig. 1:**
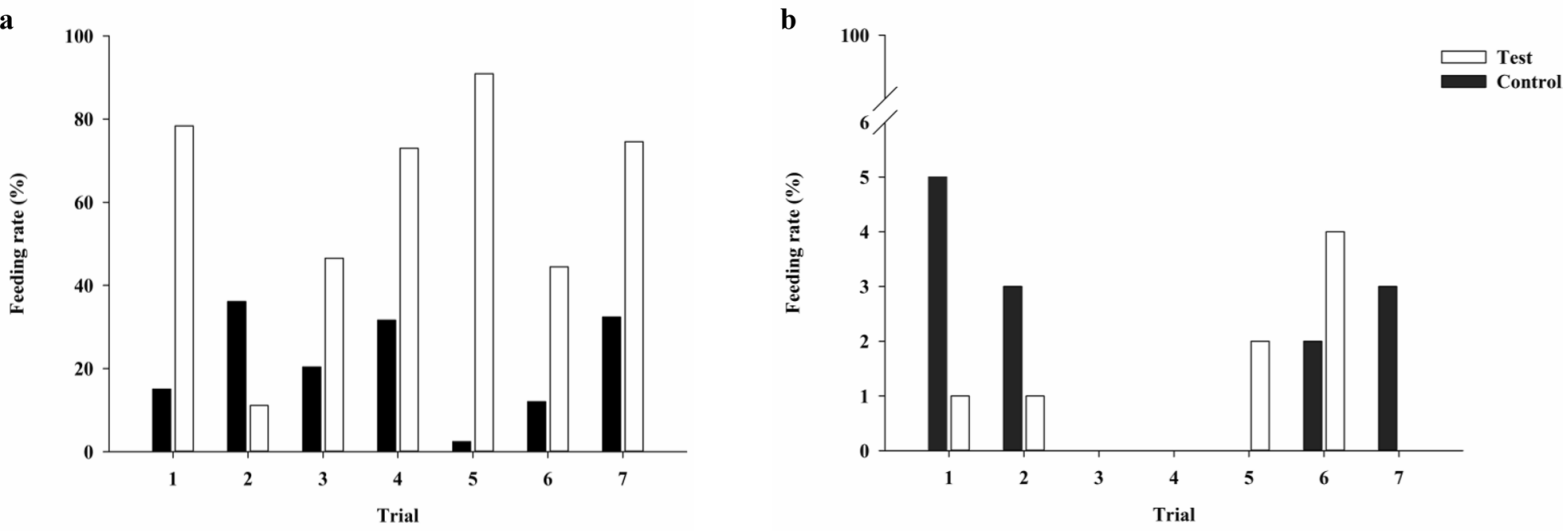
**a, b** Feeding rate (percentage of engorged phlebotomines) in the seven paired trials comparing the test (pig small intestine; white bars) with the control (chick skin; black bars) membrane. Laboratory-bred *Lutzomyia longipalpis* (**a**) and field-caught *Nyssomyia antunesi* (**b**)

A total of seven comparative trials were performed for laboratory-bred and field-caught phlebotomines. In all seven comparative trials with *L. longipalpis*, the feeding rate with the test membrane was significantly greater than with the control (*t*-test, *t* = -3.3860, *P* = 0.0054). In contrast, the feeding rate of *N. antunesi* was greater with the control than with the test membrane; however, the difference was not statistically significant (*t*-test, *t* = 0.7746, *P* = 0.4535) (Fig. 1).

**Fig. 2:**
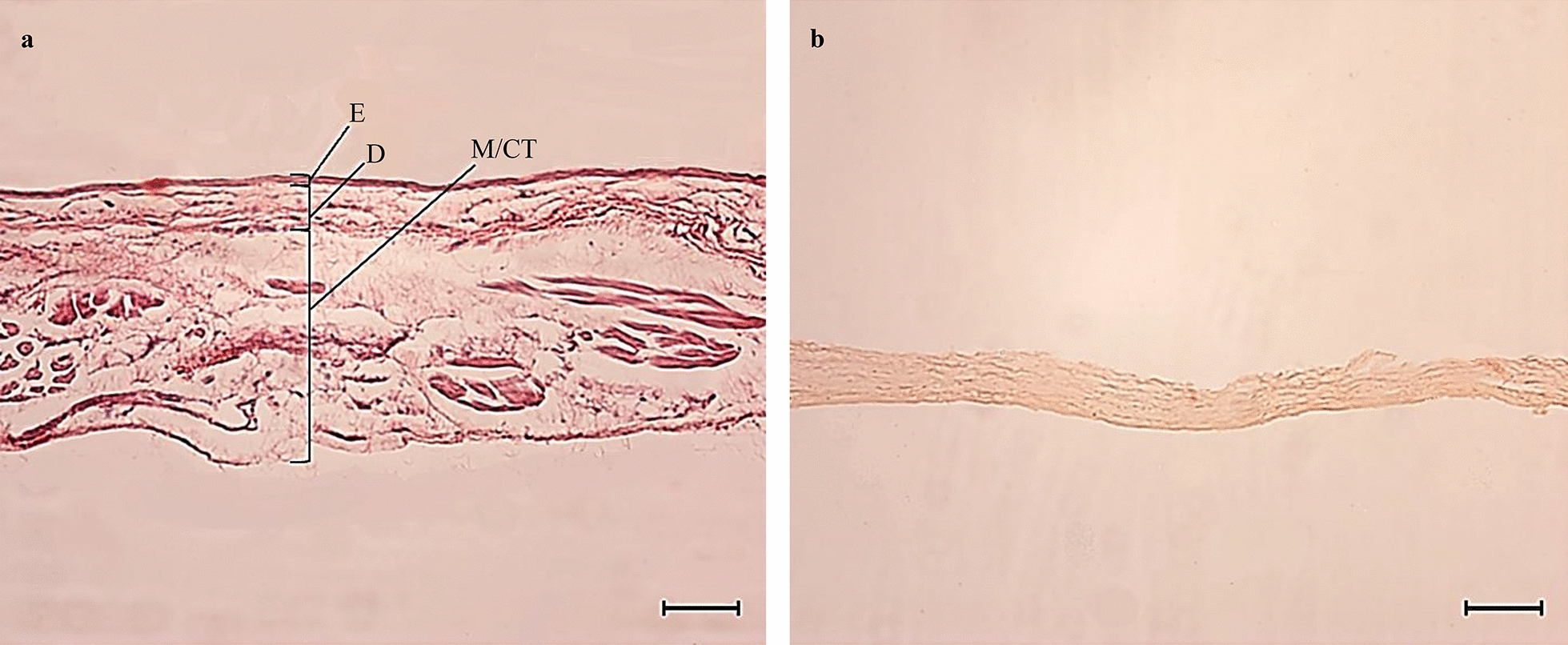
**a, b** Photomicrographs of haematoxylin–eosin stained histological sections of the membranes. **a** Chick skin showing sublayers, **b** industrially processed pig small intestine showing no tissue differentiation. Scale bar = 50 µm. *E* Epidermis, *D* dermis, *M*/*CT* muscle/connective tissue

The average thickness and SD were 184 ± 83 µm for the control and 34 ± 12 µm for the test membrane, and the difference was statistically significant (Mann–Whitney* U*-test, *U* = 0.00, *Z* = 2.8823, *P* = 0.0039). The histology of the two types of membrane differed due to the different functions of their tissues. The basic structure of the epidermis, dermis and muscle/conjunctive tissue was observed for all the sections of chick skin. In contrast, histological examination of the pig small intestine sections showed a uniform band with no cellular distinction or visible tissue layers (Fig. 2).

There were no significant relationship between the feed rate of laboratory-bred *L. longipalpis* and membrane thickness for the control (Pearson’s correlation coefficient, *r* = -0.0947, *P* = 0.8584) or test membrane (Pearson’s correlation coefficient, *r* = 0.7989, *P* = 0.0566). A moderate positive correlation was observed in all cases between the number of phlebotomines that fed and the total number of phlebotomines in the cage for the control and test membranes in the trials with *L. longipalpis* (Pearson’s correlation coefficient, *r* = 0.5788, *P* = 0.1733 and *r* = 0.6002, *P* = 0.1541, respectively) and in those with *N. antunesi* (Pearson’s correlation coefficient, *r* = 0.4684, *P* = 0.2891 and *r* = 0.6313, *P* = 0.1283, respectively).

## Discussion

The general feeding rate of *L. longipalpis* was high, which is in agreement with other studies [15, 39], but contrasts with results obtained with other species [15, 16, 20, 40]. In the paired trials, the feeding rate was greater with the test membrane than with the control membrane. These results may be explained by several factors. Firstly, standard conditions for membrane feeding with chick skin generally include overnight exposure [16], thus the short duration of feeding (2 h) and/or daytime feeding in the paired trials may have been insufficient/unsuitable for blood-feeding through chick skin. Secondly, the frequent moistening of the membranes may have disturbed the phlebotomines. Thus, the phlebotomines may have needed a longer feeding duration than that used in this study (2 h) to successfully feed through the chick skin, and it may have taken time for them to readapt and resume feeding following the disturbance of their environment caused by membrane moistening. Thirdly, attraction to a membrane may also be triggered by spatial and/or olfactory memory [15, 41].

The general feeding rates of the field-caught species were low. Additionally, there was no difference in feeding rate of *N. antunesi* between the control and test membranes in the paired trials. Data on *N. antunesi* are noteworthy because of its medical importance as a suspected vector of *Leishmania lindenbergi* in Belém and particularly in the area sampled, where it is well known as a dominant species [28]. Membrane feeding is generally unattractive for field-caught phlebotomines, even when the skin that is used is from their recognized hosts [18]. According to Harre et al. [17], females of recently established colony strains are often reluctant to artificially feed on blood, which demonstrates how challenging the maintenance of phlebotomines under laboratory conditions is. Despite the low rates recorded, a greater number of phlebotomine species fed through the test membrane compared with the control membrane.

It was not possible to accurately determine the status of the field-caught specimens. However, this factor could have had a major impact on the results where the female sand flies were concerned. The ecotopes of the capture sites usually contain resting females that bite aggressively when disturbed [28], but teneral specimens would also have been present, which are unable to feed. Due to technical limitations at the time of sampling, it was not possible to screen the insects according to life stage and thus avoid this type of biological bias.

The structure of the control membrane was similar to that reported by Couteaudier and Denesvre [42] and Umar et al. [36]. The histology of the test membrane was in agreement with that reported by Koolmees et al. [32]; however, only the submucosal layer of the small intestine was examined since the other layers—mucosal, serous and muscular—were likely eliminated during the intestinal cleaning process that is commonly performed in the industrial production of natural casings for sausages. For example, Wijnker et al. [43] observed the elimination of 90% of the mucosa and 48% of the lymphoid tissue through the latter process.

Although there were significant differences in the average thicknesses of the control and the test membranes, there was no correlation between membrane thickness and feeding rate for *L. longipalpis*. This suggests that other variables may be associated with the blood-feeding performance of phlebotomines, such as the factors discussed above which are related to the biology of the insects. On the other hand, a moderate positive correlation was observed in all cases between the number of phlebotomines that fed and the total number of phlebotomines in the cage for each type of membrane and for each species. Synergism may arise when several females feed at that same time, e.g. Triped et al. [44] demonstrated that cooperative feeding in *L. longipalpis* maximizes the effects of inoculated salivary components in the host which facilitates blood intake and counteracts host immune responses.

Further studies are needed to ascertain if *L. longipalpis* colonies can be maintained using artificial feeding systems comprising processed pig intestine sausage casing. We also suggest that a broader array of industrially processed membranes from different sources should be compared to ensure reproducibility.

## Conclusions

The results presented here indicate that the revised artificial blood-feeding system for phlebotomines presented here has the following advantages. Firstly, it enables the setting up of a short-term experiment under well-controlled conditions, which possibly increase the viability of *Leishmania* for experimental infections. Secondly, feeding phlebotomines in a nylon cage reduces the likelihood of accidental releases, and thus increases experimental safety. Thirdly, industrially processed membranes can be accessed at low cost, are ethically acceptable, reliable and conserve well when stored for a long period of time. Seeking to refine procedures to reduce the number of animals, and/or replace them, for experimental use is a guiding principle and goal of the biomedical research community.

## Data Availability

All data supporting the conclusions of this article are included in the article. The datasets used and/or analysed during the current study are available from the corresponding author upon reasonable request.

## References

[1] Ready PD (2013). Biology of phlebotomine sand flies as vectors of disease agents. Annu Rev Entomol.

[2] Young DG, Duncan MA (1994). Guide to the identification and geographic distribution of *Lutzomyia* sand flies in Mexico, the West Indies, Central and South America (Diptera: Psychodidae). Mem Am Entomol Inst..

[3] Rowton ED, Dorsey KM, Armstrong KL (2008). Comparison of in vitro (chicken-skin membrane) versus in vivo (live hamster) blood-feeding methods for maintenance of colonized *Phlebotomus papatasi* (Diptera: Psychodidae). J Med Entomol.

[4] Bates PA, Crampton JM, Beard CB, Louis C (2012). Infection of phlebotomine sandflies with *Leishmania*. The molecular biology of insect disease vectors: a methods manual.

[5] Boelaert M, Sundar S (2014). Leishmaniasis. Manson’s tropical. Infect Dis.

[6] Jansen AM, Roque ALR (2010). Domestic and wild mammalian reservoirs. American trypanosomiasis.

[7] Roque ALR, Jansen AM (2014). Wild and synanthropic reservoirs of *Leishmania* species in the Americas. Int J Parasitol Parasites Wildl.

[8] Pimenta PF, Vanesa CF, Carolina CM, Ana Clara MA, Pires Nágila FCS., Rangel EF, Shaw JJ (2018). Biology of the *Leishmania*-sand fly interaction. Brazilian sand flies: biology, taxonomy, medical importance and control.

[9] Tesh RB, Modi GB (1984). A simple method for experimental infection of phlebotomine sand flies with *Leishmania*. Am J Trop Med Hyg.

[10] Ticha L, Kykalova B, Sadlova J, Gramiccia M, Gradoni L, Volf P (2021). Development of various *Leishmania* (Sauroleishmania) *tarentolae* strains in three *Phlebotomus* species. Microorganisms.

[11] Fatemi M, Saeidi Z, Noruzian P, Akhavan AA (2018). Designing and introducing a new artificial feeding apparatus for sand fly rearing. J Arthropod-Borne Dis.

[12] Lawyer PG, Meneses C, Rowland T, Rowton ED (2016). Care and maintenance of phlebotomine sand flies.

[13] Adler S, Theodor O (1927). The behavior of cultures of *Leishmania* sp. *Phlebotomus papatasi*. Ann Trop Med Parasitol.

[14] Paiva BRD, Secundino NFC, Pimenta PFP, Galati EAB, Andrade JHF, Malafronte RDS (2007). Padronização de condições para detecção de DNA de* Leishmania* spp. em flebotomíneos (Diptera, Psychodidae) pela reação em cadeia da polimerase. Cad Saude Publica..

[15] Ready PD (1978). The feeding habits of laboratory-bred *Lutzomyia longipalpis* (Diptera: Psychodidae). J Med Entomol.

[16] Ward RD, Lainson R, Shaw JJ (1978). Some methods for membrane feeding of laboratory reared, Neotropical sandflies (Diptera: Psychodidae). Ann Trop Med Parasitol.

[17] Harre JG, Dorsey KM, Armstrong KL, Burge JR, Kinnamon KE (2001). Comparative fecundity and survival rates of *Phlebotomus papatasi* sandflies membrane fed on blood from eight mammal species. Med Vet Entomol.

[18] Mann RS, Kaufman PE (2010). Colonization of *Lutzomyia shannoni* (Diptera: Psychodidae) utilizing an artificial blood\feeding technique. J Vector Ecol.

[19] Schmidt ML (1964). Laboratory culture of two *Phlebotomus* species, *P. papatasi* and *P. orientalis*. Bull World Health Organ..

[20] Gemetchu T (1976). The biology of a laboratory colony of *Phlebotomus longipes* Parrot & Martin (Diptera: Phlebotomidae). J Med Entomol.

[21] Cabrera OL, Munstermann LE, Cárdenas R, Gutiérrez R, Ferro C (2002). Definición de las condiciones de temperatura y almacenamiento adecuadas en la detección de ADN de* Leishmania* por PCR en flebotominos. Biomedica.

[22] Noguera P, Rondón M, Nieves E (2006). Effect of blood source on the survival and fecundity of the sandfly *Lutzomyia ovallesi* Ortiz (Diptera: Psychodidae), vector of *Leishmania*. Biomedica.

[23] Cabrera OL, Munstermann LE, Cárdenas R, Ferro C (2003). PCR para la confirmación de transmisión experimental de *Leishmania chagasi* a hámster sano por picadura de *Lutzomyia longipalpis* (Diptera: Psychodidae). Biomedica.

[24] Costa-da-Silva AL, Carvalho DO, Kojin BB, Capurro ML (2014). Implementation of the artificial feeders in hematophagous arthropod research cooperates to the vertebrate animal use replacement, reduction and refinement (3Rs) principle. J Clin Res Bioeth..

[25] Lawyer P, Killick-Kendrick M, Rowland T, Rowton E, Volf P (2017). Laboratory colonization and mass rearing of phlebotomine sand flies (Diptera, Psychodidae). Parasite.

[26] Chagas AC, Medeiros JF, Justiniano SCB, Pessoa FAC (2007). Haematophagic behavior in laboratory of *Lutzomyia cruzi* (Mangabeira) (Diptera: Psychodidae) in relation to three mammalian blood sources in Manaus. Brazil Acta Amaz.

[27] Munstermann LE, Marquardt WH (2004). Care, maintenance, and experimental infection of phlebotomine sand flies. Biology of disease vectors.

[28] Sánchez Uzcátegui YDV, Vasconcelos Dos Santos T, Silveira FT, Ramos PK, Dos Santos EJM, Póvoa MM (2020). Phlebotomines (Diptera: Psychodidae) from a urban park of Belém, Pará State, northern Brazil and potential implications in the transmission of American cutaneous leishmaniasis. J Med Entomol..

[29] McMahon JP (1968). Artificial feeding of *Simulium* vectors of human and bovine onchocerciasis. Bull World Health Organ.

[30] Galati EA, Rangel EF, Shaw JJ (2018). Phlebotominae (Diptera, Psychodidae): classification, morphology and terminology of adults and identification of American taxa. Brazilian sand flies: biology, taxonomy, medical importance and control.

[31] Pennington P, Beard CB, Anderson J, Marquardt WH (2004). Care, maintenance, and handling of infected triatomines. Biology of disease vectors.

[32] Koolmees PA, Tersteeg MHG, Keizer G, Van Den Broek J, Bradley R (2004). Comparative histological studies of mechanically versus manually processed sheep intestines used to make natural sausage casings. J Food Prot.

[33] Da Silva DT, Matos PS, Lima AM, Furtado AP, Hamoy I, Matos ER (2018). *Ellipsomyxa arariensis* n. sp. (Myxozoa: Ceratomyxidae), a new myxozoan parasite of *Pygocentrus nattereri* Kner, 1858 (Teleostei: Characidae) and *Pimelodus ornatus* Kner, 1858 (Teleostei: Pimelodidae) from Marajó Island, in the Brazilian Amazon region. Parasitol Res..

[34] Da Silva Ferreira RL, Da Silva DT, De Carvalho AA, Bittencourt LS, Hamoy I, Matos E (2021). *Ellipsomyxa tucujuensis* n. sp. (Myxozoa: Ceratomyxidae), a parasite of *Satanoperca jurupari* (Osteichthyes: Cichlidae) from the Brazilian Amazon. Parasitol Int..

[35] Luna LG (1968). Manual of histologic staining methods of the Armed Forces Institute of Pathology.

[36] Umar AA, Bashir N, Atabo SM (2020). Comparative skin histology of Fulani ecotype and broiler chickens (*Gallus gallus domesticus*) in Sokoto State Nigeria. IJAPR.

[37] Röhe I, Hüttner FJ, Plendl J, Drewes B, Zentek J (2018). Comparison of different histological protocols for the preservation and quantification of the intestinal mucus layer in pigs. Eur J Histochem.

[38] Ayres M, Junior Ayres M (2000). BioEstat 20: aplicações estatísticas nas áreas das ciências biológicas e médicas.

[39] Killick-Kendrick R, Leaney AJ, Ready PD (1977). The establishment, maintenance and productivity of a laboratory colony of *Lutzomyia longipalpis* (Diptera: Psychodidae). J Med Entomol.

[40] Hertig M, McConnell E (1963). Experimental infection of Panamanian *Phlebotomus* sandflies with *Leishmania*. Exp Parasitol.

[41] Freitas JSD, Reinhold-Castro KR, Casanova C, Silva JPD, Previdelli I, Teodoro U (2009). Memória espacial e/ou olfativa em flebotomíneos em área endêmica de leishmaniose tegumentar americana, sul do Brasil. Rev Soc Bras Med Trop.

[42] Couteaudier M, Denesvre C (2014). Marek’s disease virus and skin interactions. Vet Res.

[43] Wijnker JJ, Tersteeg MHG, Berends BR, Vernooij JCM, Koolmees PA (2008). Quantitative histological analysis of bovine small intestines before and after processing into natural sausage casings. J Food Prot.

[44] Tripet F, Clegg S, Elnaiem DE, Ward RD (2009). Cooperative blood-feeding and the function and implications of feeding aggregations in the sand fly, *Lutzomyia longipalpis* (Diptera: Psychodidae). PLOS Negl Trop Dis.

